# The meaning of the MS Degree in Medical Physics, Part 2

**DOI:** 10.1120/jacmp.v15i2.4932

**Published:** 2014-03-06

Along with many of you, I read and followed with great interest the “MS vs. Ph.D. for Residency” topic on the AAPM Bulletin Board. My impression considering all of the contributions through January 31 is that there was general consensus that the trends I mentioned in the previous editorial (Part 1) are valid. Most everyone agrees there are too many CAMPEP MS graduates. In addition, a large number of residency programs are reserved for PhD students only and many of these are open to non‐CAMPEP PhD graduates through the alternative pathway. The responses consisted largely of complaints, but there were a few helpful observations:

- A CAMPEP restriction on the number of academic program graduates might not stand in court
- Some residency programs face institutional restraints to admit only PhD residents
- Degree creep is expected for professions associated with lucrative compensation
- Note that even Universities do not require a PhD for doctors (physicians)
- CAMPEP MS programs should create CAMPEP residency positions for their graduates
- Aspiring physicians don't complete medical school only to find there are no internship and residency vacancies available to them
- A Master's with ABR cannot work at many University hospitals, while a PhD without ABR can
- The residency GME offices at university hospitals look at the medical physics field and, in the absence of an MD degree, have decided the PhD is the terminal degree
- The CAMPEP didactic training requirements do not vary much between PhD or MS
- The medical community is a world of doctors and more specifically a world of Professional Doctorates

I want to make it clear this list was posted by others in response to the editorial; I offer my thanks to those who shared these thoughts. I also want to thank George Starkschall for offering the following list of activities the AAPM is taking to address the problem of too many MS students (personal correspondence, January 23, 2014):

• CAMPEP requires that every graduate program posts on a publically‐accessible website their program's track record in placement of their graduates
• In order to create more residency positions, the AAPM and RSNA are jointly funding an initiative in which they are providing 50% stipend support to new imaging physics residency programs
• CAMPEP is providing on its website a sample Self‐Study and business plan for private practices who wish to apply for accreditation of their residency programs
• AAPM has held, and is planning to continue to hold, workshops to assist potential residency program directors in the development of Self‐Studies, with special focus on hub‐and‐spoke programs that would involve private practices
• Recognizing that there will remain more students in MS programs than there are clinical job openings, the Students and Trainees Subcommittee of the AAPM is preparing a guide to non‐clinical employment in medical physics

Consider the following professionals: physicians, dentists, podiatrists, optometrists, psychologists, pharmacists and audiologists. These folks all complete professional doctorate programs that consist of both academic courses and clinical competencies. When they graduate, they are fully qualified to practice, treat, and bill patients. Now consider these allied health workers: occupational therapists, physical therapists, nurses, radiation therapists, nuclear medicine technologists, dental hygienists, and speech language pathologists. These professionals complete BS and MS programs that also embrace both academic courses and clinical competencies. When they become program graduates, they are fully qualified to practice and treat patients, although they usually bill for services provided by the facility, depending on the employment arrangement. What both of these groups have in common is that the programs are professional and unified.

So why would medical physics operate under the radical model of separating into two programs the academic and residency portion of our training? Does this model benefit patients, employers, physician colleagues or the students our profession would attract in some unique manner? Additionally, the power to name our training lies with us. A master's degree plus a residency is the same training as a professional doctorate. Why would we choose to name this training deliberately to exclude our students from the professional staffs of many universities and academic centers? Indeed, in some centers, medical physicists cannot be trained as residents unless they possess a PhD.

It must be mentioned that many MS programs face huge hurdles if they wish to convert to DMP programs. Many do not have the resources to provide a residency experience to their students, and many others would face insurmountable challenges getting a professional doctorate program approved within the university and state academic processes.

It was pointed out that after the 33 or so residency positions reserved for PhD graduates are removed from consideration, that MS graduates have great success competing for the remaining slots. However, for these 33 positions, someone made the decision not to consider MS graduates in the first place; this is the trend I see. While a few MS programs that incorporate significant clinical training in their schedule are having great success seeing their graduates find residency slots, including their own, there are other programs whose entire graduating class was collectively unable to land a single residency position. In spite of this, there are a large number of new academic MS programs seeking and receiving CAMPEP accreditation each year.

**Table 1:** CAMPEP statistics from 2012

	*2009*	*2010*	*2011*	*2012*
# Programs offering MS/MSc Degrees	28	31	32	39
# Programs offering PhD Degrees	21	22	24	29
# Programs offering DMP Degrees				1
Enrollment MS/MSc	333	431	450	466
Enrollment PhD	345	458	465	534
DMP				20
# Graduates MS/MSc Degrees	147	168	148	198
# Graduates PhD Degrees	63	68	67	80
# Graduates DMP				4
# Entering Residency MS/MSc	15	20	33	44
# Entering Residency PhD	10	22	23	24

Per Halvorsen, Chair of the Professional Council, shared this thought with me: “The meaning of this problem is that there is a mismatch between institutional and professional needs and priorities. It seems fairly clear that many residency programs are accepting PhDs exclusively or giving very strong bias toward PhD applicants based not on what the profession needs in terms of well‐rounded clinical physicists, but on what that institution needs in terms of its research program. If this is as pervasive as the thread seems to imply, then we may indeed have a problem with the overall ‘system’ of preparing new clinical physics professionals.”

The latest information I have from CAMPEP shows that the trend toward oversupply of MS training programs and students is expanding. This information was graciously supplied by Dr. Ed Jackson (personal correspondence, January 14, 2014).

Consider the following scenario. A large university center faced with new financial realities decides to downsize its Qualified Medical Physicist (QMP) staff in half — eliminating those it considers the least productive. Then it monitors the workload of the remaining physicists and adds physicist assistant positions one at a time. The new hires are the MS physicists who cannot land residency positions; the salary is less than half what they were paying for a QMP and two‐thirds that of a medical dosimetrist. When the workload is acceptable in the eyes of administration, it stops hiring. Thereafter, when a QMP vacates a position, the replacement is a non‐QMP who is grateful to have a job in the industry at between fifty and sixty thousand dollars per year. Without licensure and an associated legally defined scope of practice, there is no remedy to this eventuality. Is this the future of the profession?

Consider the dignity of that bright potential MS student who wishes to join you as your colleague. To be specific, we are talking about the opportunity to join you as a practicing professional specialist or expert — that is, as a QMP. Are you asking him to borrow twenty to forty thousand dollars for a one‐in‐four chance of entering the profession? Why would you do that when the competing medical professions offer a 100% chance of entry upon successful completion of a program? Yes, I hear the chorus of defenders of this arrangement saying no physician is guaranteed a radiation oncology residency. This disingenuous argument fails as follows: While a first‐choice residency is not guaranteed, the physician is not prevented from competing for any other residency; MS graduates do not have the luxury of considering other types of residencies. Additionally, while the physician is not prevented from practicing medicine, that physician cannot practice radiation oncology and is not a threat to take away a position now occupied by a radiation oncologist or undermine their job security and lifestyle.

So what is the end result of our offer? Come join us as students and, if you are one of the chosen few, you may join us for a season and experience the undermining of our profession and the erosion of your lifestyle by your student colleagues who were not among the chosen? And if you are not successful, you can still join us in a lower non‐professional capacity at much lower compensation and participate in the great leveling of the profession? I would love to hear a response to these questions that supports and affirms the dignity of our students, while also affirming the dignity of those who practice as our professional colleagues. Whatever answer is forthcoming will define the meaning of the MS degree in Medical Physics.

Michael D. Mills, PhD

Editor‐in‐Chief

